# Potential risk of heartworm infection in the United States of America and its projected incidence by 2100

**DOI:** 10.1186/s13071-026-07293-5

**Published:** 2026-08-20

**Authors:** Rodrigo Morchón, Iván Rodríguez-Escolar, Manuel Collado-Cuadrado, Elena Infante González-Mohino, José Alberto Montoya-Alonso, Elena Carretón, Alfonso Balmori-de la Puente

**Affiliations:** 1 Zoonotic Diseases and One Health GIR, Biomedical Research Institute of Salamanca (IBSAL), Centro de Estudios Ambientales y Dinamización Rural (CEADIR), Faculty of Pharmacy, University of Salamanca, Salamanca, Spain; 2 Internal Medicine, Faculty of Veterinary Medicine, Research Institute of Biomedical and Health Sciences, University of Las Palmas de Gran Canaria, Las Palmas, Spain

**Keywords:** Heartworm disease, *Dirofilariaimmitis*, *Aedesvexans*, *Aedesalbopictus*, Ecological niche model, USA

## Abstract

**Background:**

Heartworm disease, caused by the parasitic roundworm *Dirofilaria immitis*, is a vector-borne zoonosis transmitted by culicid mosquitoes and widely distributed in the USA, where prevalence is highest in the southeastern states. Transmission dynamics are influenced by environmental and anthropogenic factors, and climate change is expected to modify its distribution. Within a One Health framework, this study generates monthly and annual risk maps for heartworm transmission in the USA and projects the habitat suitability of vectors to the year 2100.

**Methods:**

Ecological niche models were constructed for *Aedes albopictus* and *Aedes vexans* using MaxEnt and occurrence data from the Global Biodiversity Information Facility (GBIF). Bioclimatic variables from WorldClim and environmental layers related to hydrology, vegetation, and human activity obtained from free global datasets were selected after multicollinearity testing. Growing degree days (GDD) were calculated to estimate monthly and annual *D. immitis* generations using CHELSA (Climatologies at high resolution for the Earth's land surface) temperature data. Habitat suitability models were combined with parasite generations to produce infection risk maps for the USA, validated against clinical case data. Future projections to 2100 were generated under the RCP (Representative Concentration Pathways) 8.5 scenario.

**Results:**

Our results indicate that the highest risk of infection is concentrated in the southeastern states and Gulf Coast, where conditions are warmer and more humid, at least during certain periods of the year. In contrast, risk decreases towards the west, particularly in the Rocky Mountains, Sierra Nevada, and the deserts of Arizona and Nevada. Regarding seasonal variation, infection risk remains very low across most of the USA during the winter months, gradually increasing month by month and peaking in summer. Finally, projections for 2100 suggest a rise in infection risk, particularly in Midwest and Northeast regions and higher-altitude areas.

**Conclusions:**

Heartworm transmission risk in the USA shows strong geographical expansion potential driven by climate conditions. Our risk maps reveal emerging areas of suitability and provide an early warning tool for One Health surveillance and prevention strategies.

**Graphical Abstract:**

**Figure:**
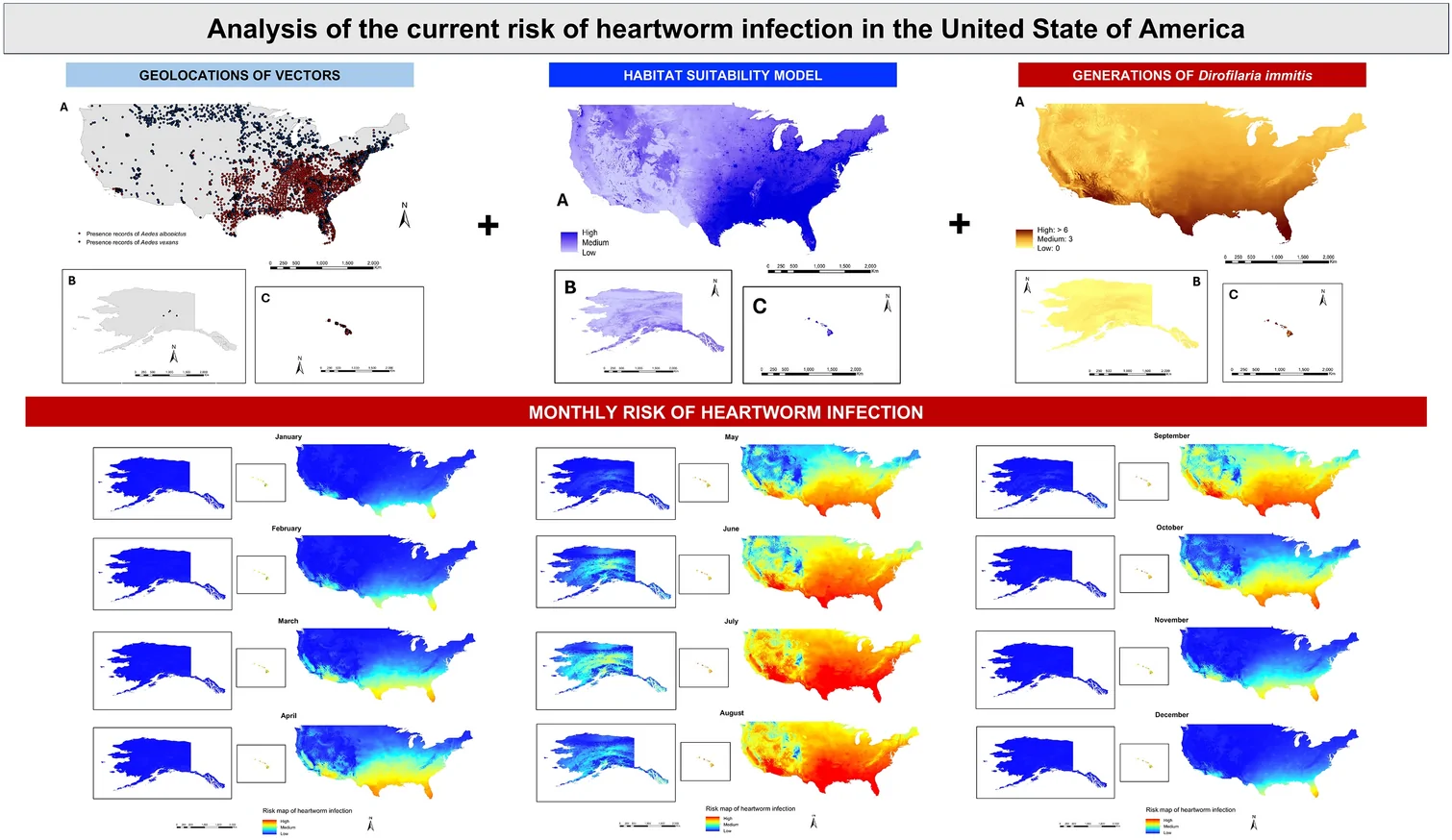

**Supplementary Information:**

The online version contains supplementary material available at 10.1186/s13071-026-07293-5.

## Background

Heartworm disease, caused by the parasitic roundworm *Dirofilaria immitis*, is a vector-borne zoonosis whose most important definitive hosts are canids, with domestic dogs serving as the main known reservoir. Other carnivores such as felids and mustelids [1, 2] can also be affected, as well as humans, who are considered accidental hosts [3]. Several culicid mosquito species from the genera *Aedes*, *Anopheles*, *Culex*, and *Mansonia*, among others, act as vectors of the infection [4, 5]. In the United States of America (USA), *Ae. albopictus* and *Aedes vexans* are among the most important mosquito species involved in heartworm transmission due to their wide distribution in high-incidence regions [6].

In the definitive host, adult *D. immitis* worms are located between the pulmonary artery and the right ventricle of the heart. They release microfilariae into the bloodstream, which are ingested by mosquito vectors during blood-feeding. Within the mosquito, microfilariae reach the infective third larval stage (L_3_) in approximately 14 days and migrate to the mosquito proboscis, where they remain until transmission to a new host. This process depends on the environmental temperature. Experimental infections have shown that at 22 °C, microfilariae develop to L_3_ in approximately 16–20 days, while at 28–30 °C, development takes 8–10 days. However, below 14 °C, larval development is interrupted, although larvae remain viable for up to 3 days [7, 8]. Infective L_3_ larvae are transmitted to another host during a subsequent blood meal and undergo two additional molts to reach adulthood, finally becoming established in their typical cardiopulmonary location, where they become sexually mature.

The USA has one of the highest reported burdens of heartworm disease worldwide, with major health and economic impacts [9]. In 2022, the highest incidence was reported in the southeastern and south-central regions, with more than 50, and in some areas over 100 cases per year, whereas isolated foci were recorded in the Northeast, and the incidence was nearly residual in the West [10]. Reported prevalence ranges from 2.6% to 6.3% in domestic dogs and around 0.3% in cats [11, 12]. The highest prevalence occurs in the southeastern USA (~7.5%), particularly in Alabama, Arkansas, Georgia, Florida, Louisiana, Mississippi, Tennessee, Texas, and South Carolina. However, other states such as Minnesota (7.7%), Iowa (12.5%), Illinois (10.5%), Virginia (9.1%), and Colorado (5.3%) also show significant prevalence. In contrast, northeastern states generally report negligible prevalence, except for Connecticut (16.7%) and, to a lesser degree, New York (4.2%) [12]. Similar prevalence rates have been reported in shelter dog studies in the eastern USA, with overall prevalence of 11.2%, with the highest values in the southeast (17.4%), followed by 5.3% in the Midwest and 1.6% in the Northeast [13].

Several factors affect transmission risk and disease dynamics, including the size of the country, climate variability, topography, temperature, rainfall, the distribution of mosquito vectors, and the introduction of invasive species, as well as increased movement of people and pets and anthropogenic activity (e.g., urban development, irrigation, and creation of stagnant water bodies) [1, 6]. Within a One Health framework, heartworm prevention and control strategies include diagnosis and clinical management of infected animals, administration of chemoprophylaxis, and implementation of mosquito exposure reduction measures [3, 14]. Risk maps based on ecological niche modeling (ENM) have been widely used to estimate the potential distribution of vectors [15, 16], infected hosts [17–22], and vector–parasite interactions [23–28], allowing a more realistic evaluation of infection risk [29–32].

The objective of this study was to develop monthly and annual risk maps for *D. immitis* infection in the USA and evaluate potential risk scenarios using the future suitability model projections at 2100 as a tool for heartworm prevention and control for veterinary and public health professionals, pet owners, and the general population within a One Health perspective.

## Methods

### Study area

**Fig. 1:**
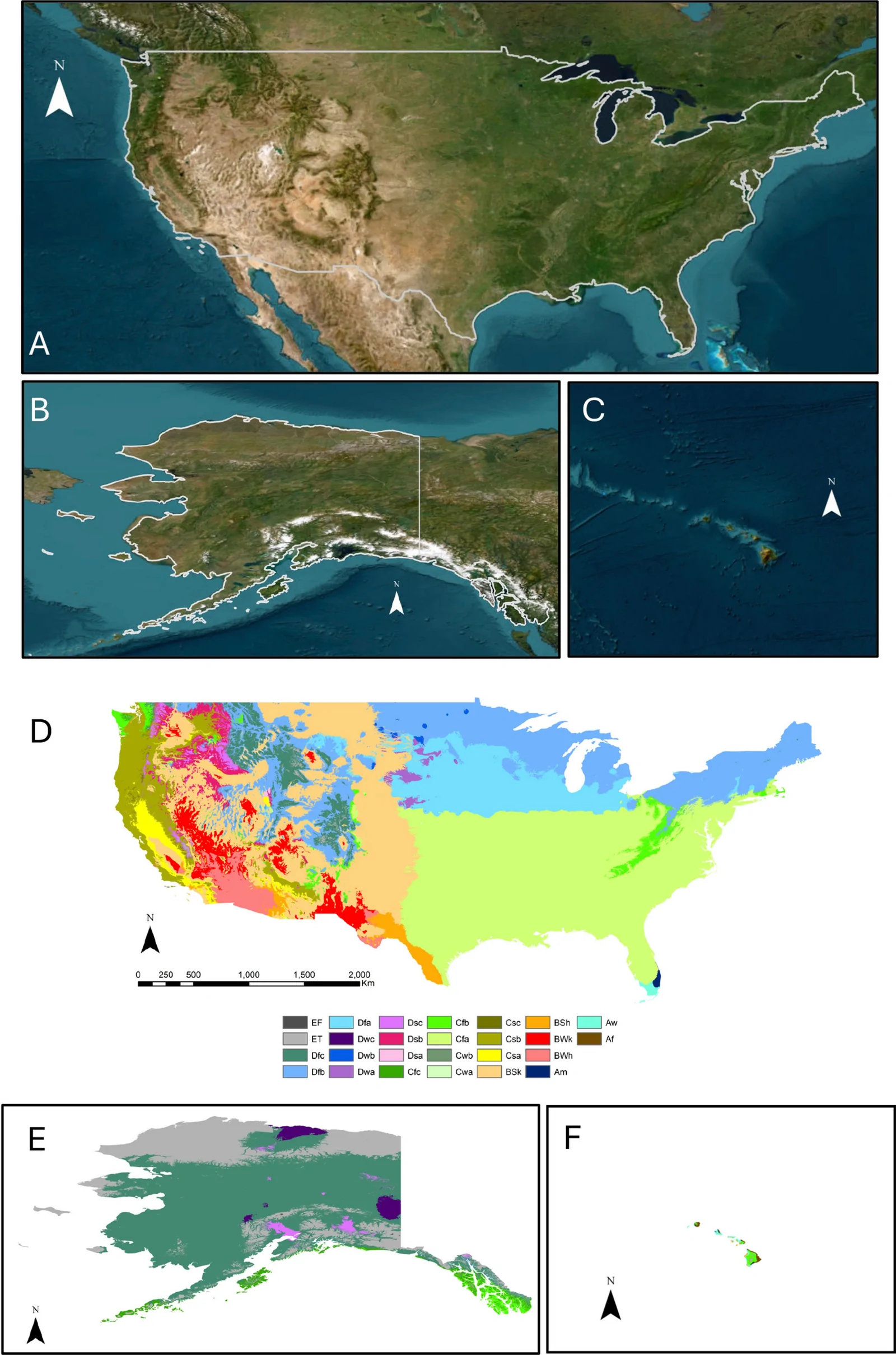
Geographical location of the United States of America (USA): Continental USA (**A**), Alaska (**B**), and Hawaii (**C**). Climates according to the Köppen Climate Classification System of the USA: Continental USA (**D**), Alaska (**E**), and Hawaii (**F**) (EF: ice cap, ET: tundra, Dfc: subarctic, Dfb: warm-summer humid continental, Dfa: hot-summer humid continental, Dwc: monsoon-influenced subarctic, Dwb: monsoon-influenced warm-summer continental, Dwa: monsoon-influenced hot-summer continental, Dsc: subarctic with dry summer, Dsb: warm-summer continental with dry summer, Dsa: hot-summer continental with dry summer, Cfb: marine West Coast (temperate oceanic), Cfc: subpolar oceanic, Cwa: monsoon-influenced humid subtropical, Cwb: subtropical highland (humid subtropical with dry winter), Csc: cold-summer Mediterranean, Csb: warm-summer Mediterranean, Csa: hot-summer Mediterranean, BSk: cold semi-arid (steppe), BSh: hot semi-arid (steppe), BWk: cold desert, BWh: hot desert, Am: tropical monsoon, Aw: tropical savanna, Af: tropical rainforest)

The USA is located in North America and covers a total area of 9,161,966 km^2^, with an estimated population of 331.8 million (Fig. 1). The country is composed of 50 states and the District of Columbia (Washington, DC), bordered by Canada to the north and Mexico to the south, and flanked by the Atlantic Ocean to the east and the Pacific Ocean to the west. In addition to the continental territory, the USA includes Alaska, located in the northwestern part of the continent and separated from Russia by the Bering Strait, and Hawaii, an archipelago of volcanic origin situated in the central Pacific Ocean. The country also has several island territories, including Puerto Rico in the Caribbean [33].

The USA exhibits a high degree of physiographical heterogeneity as a result of its vast territory. The Atlantic coastal plain in the east is characterized by lowlands and deciduous forests that rise gradually toward the Piedmont. Further inland, the Appalachian Mountains form a major orographic barrier running from northeast to southwest. West of the Appalachians, the landscape transitions into the extensive central lowlands and the Great Plains, which are traversed by the Mississippi–Missouri River system, one of the longest and most important river networks in the world. In the western part of the country, high mountain ranges dominate the topography, including the Rocky Mountains, Sierra Nevada, and the Cascade Range. These are interspersed with arid basins and major desert systems such as the Mojave, Sonoran, and Chihuahuan deserts. The highest elevations in the country are found in Alaska, including Denali (6194 m), while Hawaii is composed entirely of volcanic islands.

Climatically, the USA encompasses a broad range of climatic zones according to the Köppen classification system. The Northeast and Midwest regions experience a humid continental climate with cold winters and warm summers, whereas the southeastern states present a humid subtropical climate characterized by high temperatures and abundant rainfall. The Great Plains are dominated by semi-arid steppe climates, and the southwestern regions include extensive arid desert climates. The Pacific Coast of California has a Mediterranean climate, while the western mountain ranges exhibit alpine conditions with low temperatures year-round. Alaska experiences predominantly subarctic and polar climates, while tropical climates are present in Hawaii and southern Florida. This pronounced environmental and climatic diversity plays an important role in shaping ecological processes, including the distribution and abundance of mosquito vectors, and therefore has direct implications for the transmission risk of vector-borne diseases such as heartworm (*D. immitis*). The country is also affected by extreme weather phenomena, including hurricanes along the Gulf of Mexico and tornadoes in the central region, known as “Tornado Alley” [34, 35], which may further influence environmental stability and vector ecology.

### Presence data

**Fig. 2:**
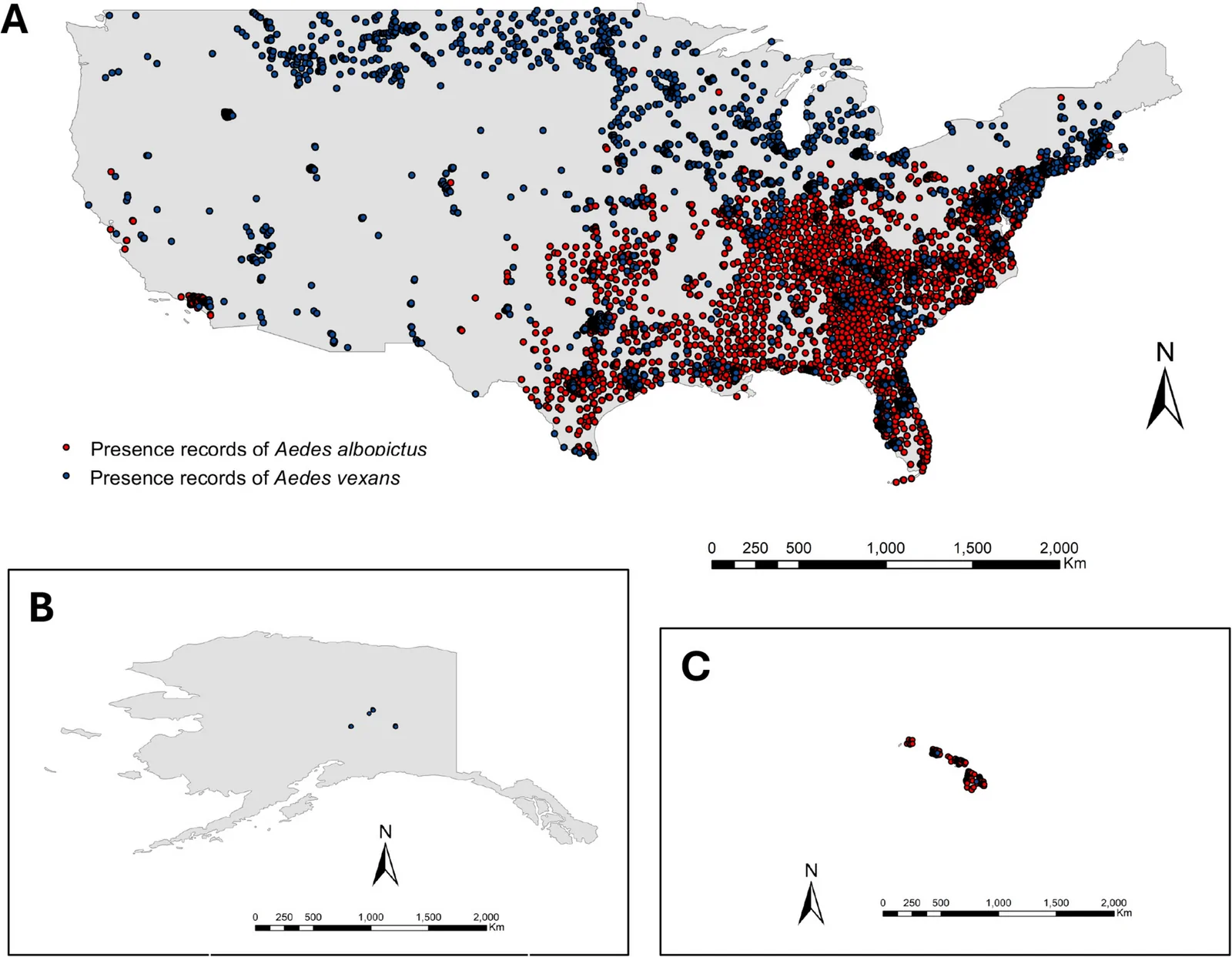
Georeferenced occurrence points of *Aedes albopictus* and *Aedes vexans* in the USA obtained from the GBIF database (GBIF.org): Continental USA (**A**), Alaska (**B**), and Hawaii (**C**)

Georeferenced occurrence records for *Ae. albopictus* (*n* = 8135) and *Ae. vexans* (*n* = 4925) were obtained from the Global Biodiversity Information Facility (GBIF) database, as these are two of the most important mosquito species involved in the transmission of dirofilariasis in the USA [6]. These species also represent those with the highest number of presence records and the best geographical coverage in GBIF [36, 37], making them suitable candidates for ENM. Other vectors mentioned in the literature were also evaluated but excluded due to lower data availability and high spatial redundancy, including *An. punctipennis* (*n* = 2788), *An. quadrimaculatus* (*n* = 2335), *Cx. pipiens* (*n* = 2039), *Ae. trivittatus* (*n* = 1622), and *Ae. canadensis* (*n* = 1354). To reduce spatial autocorrelation bias associated with clustered records, a 1 km^2^ grid was overlaid onto the dataset, retaining only one occurrence per grid cell. After data filtering and spatial thinning, a total of 5893 occurrence points were retained for *Ae. albopictus* and 3564 for *Ae. vexans* (Fig. 2).

### Bioclimatic and environmental variables

Climatic variables were obtained from the WorldClim database [38], from which 15 out of the 19 available bioclimatic variables (1970–2000) related to temperature and precipitation were downloaded, along with their projections to the year 2100 under climate change scenarios [39]. The four excluded variables (mean temperature of wettest quarter, BIO_8_; mean temperature of driest quarter, BIO_9_; precipitation of warmest quarter, BIO_18_; and precipitation of coldest quarter, BIO_19_) were removed because they combine temperature and precipitation information, which may artificially increase collinearity in ENMs.

A multicollinearity test was performed on these variables in R software [40] using Pearson’s correlation coefficient in order to identify the most informative variables, avoid cross-correlation, and improve model calibration. Variables with correlation coefficients ≥ 0.8 were excluded to reduce redundancy and prevent inflation of variable importance [41]. Following this procedure, four bioclimatic variables were selected for modeling: annual mean temperature (BIO_1_), mean diurnal range (BIO_2_), annual precipitation (BIO_12_), and precipitation seasonality (BIO_15_).

In addition to climatic layers, five environmental variables were incorporated to better characterize ecological suitability and potential distribution patterns of mosquito vectors. These variables included water bodies [42], irrigated crops [43], river networks [44], human footprint index—an integrative indicator including built environments, population density, power infrastructure, croplands, grazing lands, roads, railways and waterways—[45], and herbaceous and shrub vegetation density [46]. These variables were selected based on their potential influence on mosquito breeding, survival, and dispersal, and were obtained from freely available global environmental datasets.

All environmental layers were processed and standardized in ArcMap 10.8 [47] to ensure a consistent spatial extent (USA), resolution (1 km^2^ per pixel), and coordinate reference system (GCS_WGS_1984).

### Ecological niche models

The ENMs for *Ae. albopictus* and *Ae. vexans* were developed using the MaxEnt algorithm [48], automated through the kuenm package [41] in R [40]. MaxEnt, based on the principle of maximum entropy, estimates habitat suitability by relating species occurrence records to environmental variables, assigning probability values across the study area. Kuenm generated a total of 119 candidate models for each species by combining 17 values of the regularization multiplier M (0.1–1.0 at intervals of 0.1; 2–6 at intervals of 1; and 8 and 10), seven possible combinations of feature classes (F: linear, quadratic and product: l, q, p, lq, lp, qp, lqp), and a single set of predictor variables.

Default MaxEnt settings were used, including 10,000 random background points within the study area (pseudoabsence) and logistic output format. Model performance was evaluated based on statistical significance (partial receiver operating characteristic [ROC] < 0.05, with 100 iterations and 50% of occurrence data used for bootstrapping), omission rate (OR = 5%), and model complexity using the Akaike information criterion (AIC), using independent occurrence data (80% for training and 20% for testing). Additionally, model accuracy was evaluated with the mean area under the ROC curve (AUC) value.

The suitability maps generated for both vectors (ENM *Ae. albopictus* × ENM *Ae. vexans*) were combined with different weighting proportions (*Ae. albopictus*–*Ae. vexans* [AV]: 0–100, 10–90, 25–75, 50–50, 75–25, 90–10, 100–0). Since each raster pixel contains the probability of mosquito presence, the new combined risk value for each pixel in the resulting models was calculated using the adapted formula proposed by Balmori-de la Puente et al. [32]:

$$ENM weighted=\frac{ENMa*A+ENMv*V}{100},$$

where the habitat suitability model for *Ae. albopictus* (ENMa) and habitat suitability model for *Ae. vexans* (ENMv) are necessary, and *A and V* correspond to their relative contribution to the final model, respectively.

### Generations of *Dirofilaria immitis*

The number of *D. immitis* generations was estimated at both annual and monthly scales using the procedure described by Genchi et al. [7] and Rodríguez-Escolar et al. [29], with specific modifications implemented in a customized script. The method is based on calculating accumulated growing degree days (GDDs), defined as the number of daily mean temperature degrees exceeding 14 °C, which is the minimum thermal threshold required for parasite L_3_ to develop within the mosquito vector (extrinsic incubation phase). A generation is considered complete when a minimum of 130 GDDs are accumulated within the lifespan of the vector, estimated at a maximum of 30 days. The average numbers of generations per month and across all years were estimated to obtain monthly and annual numbers of generations in the two temporal scales. To perform these calculations within the study area, updated daily mean temperature data with a spatial resolution of 1 km^2^ per pixel were used for the period 1990–2016 [49].

Due to the large volume of data associated with the spatial extent of the study area (entire United States at 1 km^2^ resolution) and its high computational demand, all processing was carried out on the Sapphire node of the Caléndula supercomputer [50]. This module consists of 200 servers, each equipped with two Intel Xeon Platinum 646Y processors (Sapphire Rapids, 32 cores at 2.8 GHz, totaling 64 cores per server), 1 TB of RAM in 192 servers and 2 TB in eight servers, and an Infiniband NDR (Next Data Rate) interconnection at 200 Gbps. Altogether, the node provides 12,800 computing cores. Computations were run in parallel between 24the and 26th April 2025. Calculations per year required 33 cores, 100 GB, and approximately 1.5 days.

### *Dirofilaria immitis* risk map and its validation

The seven habitat suitability models for both mosquito species (*Ae. albopictus*–*Ae. vexans*; 0–100, 10–90, 25–75, 50–50, 75–25, 90–10, 100–0) were weighted using the annual number of *D. immitis* generations in equal proportion (50–50), resulting in seven average infection risk maps for the USA with different levels of vector contribution. To select the best-fit model, a validation was performed through a regression analysis between the mean infection risk predicted by each model and the average number of *D. immitis* cases per clinic in each state in 2022, according to the most recent data reported by the American Heartworm Society (AHS) [10].

The habitat suitability model that provided the average map with the highest predictive performance during validation was subsequently used to generate monthly infection risk maps. In total, 12 monthly risk maps were produced by weighting this suitability model with the monthly number of *D. immitis* generations.

### Projection to the year 2100 and rank change analysis

To project the potential distribution to the year 2100, ENMs were generated again for both vectors using MaxEnt with the same calibrated parameters as in the current period, incorporating future bioclimatic projections for 2081–2100. Climate change effects were evaluated using the HadGEM3-GC31-LL general circulation model under the RCP (Representative Concentration Pathways) 8.5 scenario, which represents a high greenhouse gas emission pathway [51, 52]. This global climate model has been widely used and validated in ecological modeling studies, as it accurately reproduces large-scale climatic patterns and has shown robust performance across different regions [53, 54].

Both the current model and the 2100 projection were converted into binary presence–absence maps using the 10th percentile training presence threshold. Finally, a range change analysis was performed using the biomod2 package in R, which calculates the percentage of grid cells gaining or losing habitat suitability in the future scenario compared with the present distribution model [55].

## Results

### Habitat suitability models for *Ae. albopictus* and *Ae. vexans*

Of the 119 habitat suitability models generated for each vector, the ones chosen were M_0.1_F_q for *Ae. albopictus* (AUC = 0.786) and M_0.3_F_lqp for *Ae. vexans* (AUC = 0.804), as they were the only models meeting the three Kuenm selection criteria. These models were subsequently used to construct suitability maps for both species with different proportions. In terms of the percentage contribution of the variables, those that contributed most to the distribution model of *Ae. albopictus* were human footprint (51.7%), followed by shrub density (20.2%) and annual mean temperature (BIO_1_) (17.1%). With regard to the distribution model for *Ae. vexans*, the variable that contributed most was human footprint (87.1%), with the rest contributing less than 4% each (Table 1).

**Table 1:** Analysis of the contribution of the 10 bioclimatic and environmental variables to the ecological niche models for *Ae. albopictus* and *Ae. vexans*

Variable	Percentage contribution	
	*Aedes albopictus* (%)	*Aedes vexans* (%)
Human footprint	51.7	87.1
Shrub density	20.2	2.9
Annual mean temperature (BIO_1_)	17.1	3.9
Precipitation seasonality (BIO_15_)	9.9	0.8
Herbaceous density	0.6	1.1
Water bodies	0.2	0.9
Irrigated crops	0.2	0.5
Rivers	0.1	0.1
Mean diurnal range (BIO_2_)	0	1.6
Annual precipitation (BIO_12_)	0	1.1

**Fig. 3:**
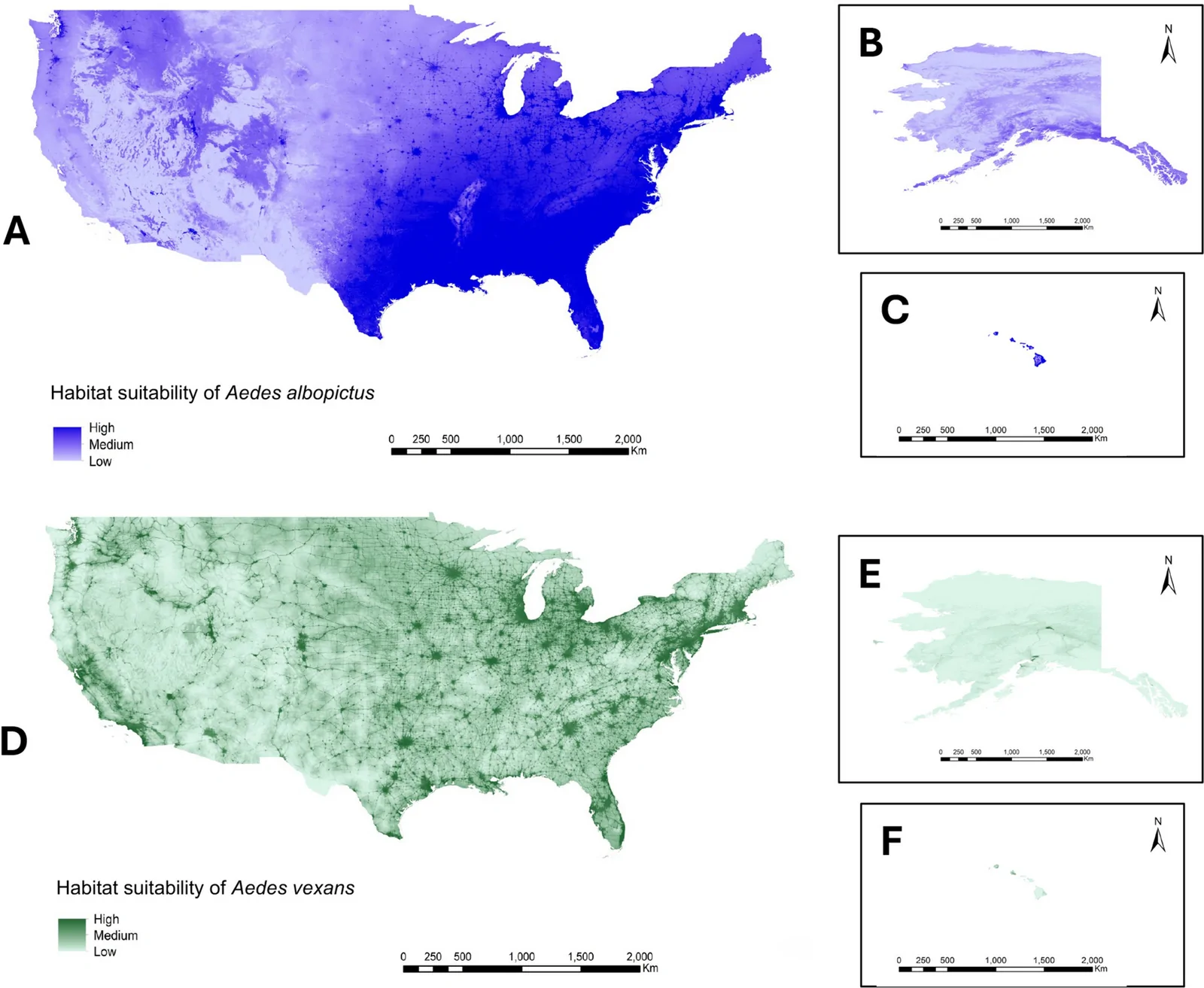
Ecological niche models showing habitat suitability for *Aedes albopictus* and *Ae. vexans* across USA territories. **A**–**C** correspond to *Ae. albopictus* in the continental USA (**A**), Alaska (**B**), and Hawaii (**C**); **D**–**F** correspond to *Ae. vexans* in the continental USA (**D**), Alaska (**E**), and Hawaii (**F**)

The suitability map for *Ae. albopictus* showed that the eastern and southeastern United States are the most favorable areas for its survival, with high suitability in the Great Lakes region, the Atlantic Coast, and the Gulf of Mexico, as well as coastal and urbanized areas (Fig. 3A–C). In contrast, suitability was low in the west and in arid inland territories. With regard to *Ae. vexans*, the most suitable habitat distribution was found in the central and eastern regions of the country, with a pattern strongly associated with urban and metropolitan areas (Fig. 3D–F).

### Number of extrinsic generations of *Dirofilaria immitis*

**Fig. 4:**
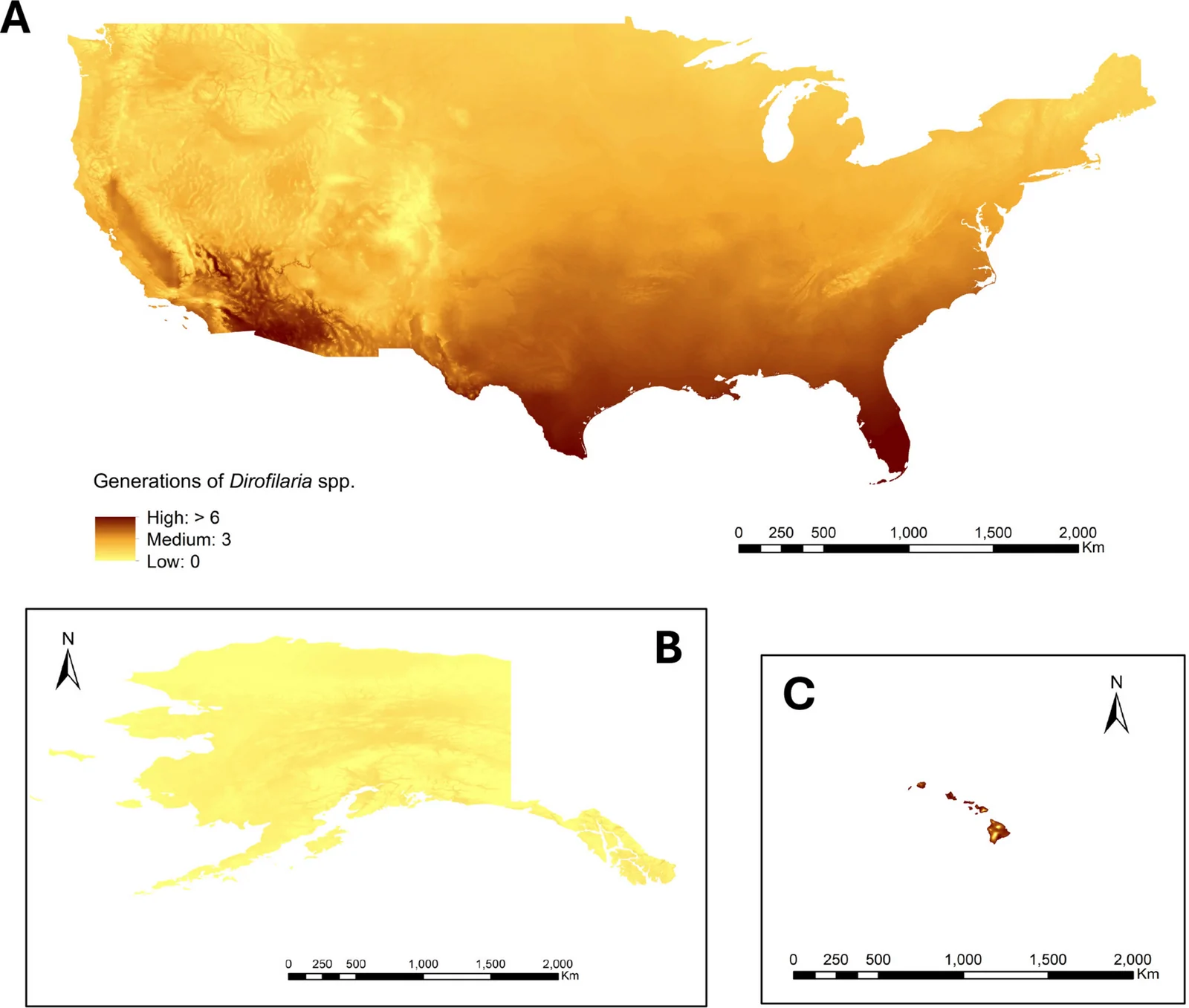
Prediction of the average number of generations of *Dirofilaria immitis* in the USA: Continental USA (**A**), Alaska (**B**), and Hawaii (**C**)

Figure 4 shows the estimated average annual number of extrinsic generations of *D. immitis* in mosquito vectors in the USA, including Alaska and Hawaii. The highest number of generations (> 6) occurred in the southeastern states, particularly along the Gulf Coast (Alabama, Florida, Louisiana, Mississippi, and Texas), as well as in Hawaii. Intermediate values (2–4 generations) were found throughout much of the central and eastern parts of the country, while, in contrast, northern regions and mountainous areas in the West showed low values (< 2 generations). In Alaska, the number of extrinsic generations is virtually zero due to low temperatures throughout the year.

With regard to the monthly maps (Supplementary Figs. 1–3), July showed the largest extent of areas with a very high number of generations (> 9), in the southeastern continental states and Hawaii, and moderate to high values in most of the center of the country. In contrast, January had the lowest values, reaching 0 generations in almost the entire territory, except for specific areas of the Gulf Coast and the Caribbean. Throughout the year, the number of generations increased progressively from January until reaching its maximum in July, following a clear latitudinal gradient, and then gradually decreased until returning to minimum values in January.

### Annual infection risk map for dirofilariasis and its validation

**Fig. 5:**
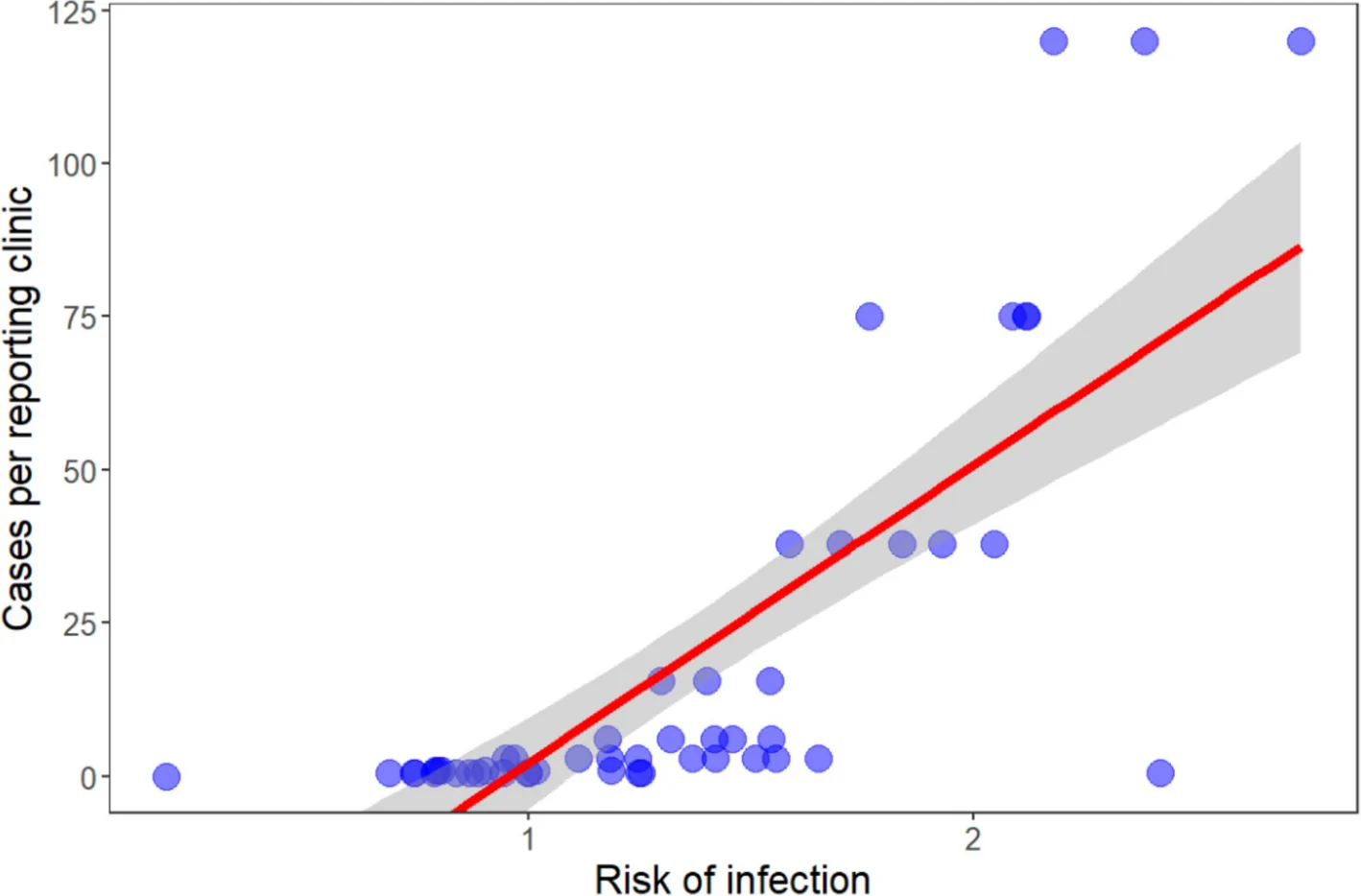
Regression plot for the validation of 100% *Aedes albopictus* risk map between the predicted mean risk of infection and the number of cases reported in each US state

**Fig. 6:**
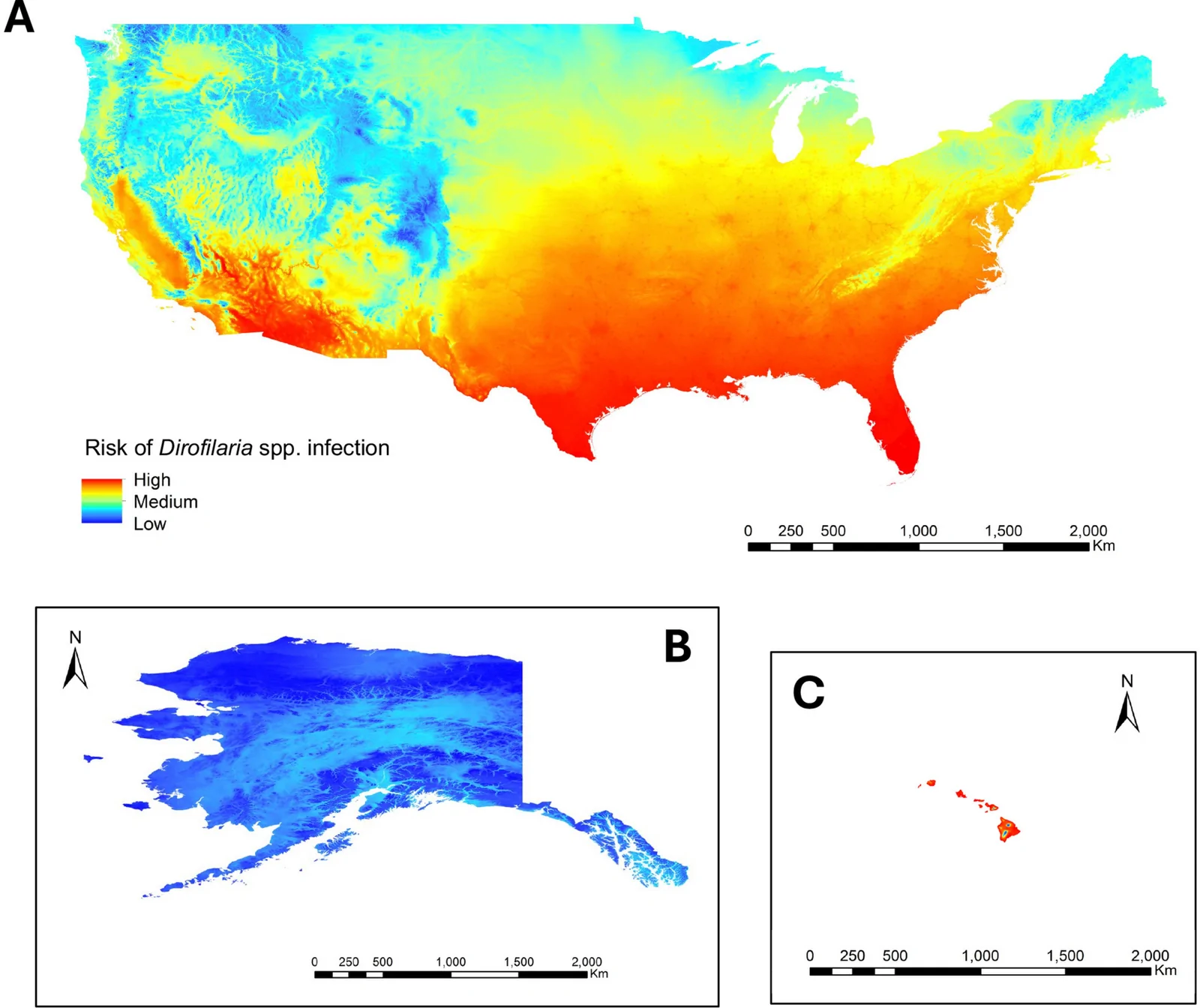
Average infection risk map for *Dirofilaria immitis* in the USA, constructed from the ecological niche model (ENM) of 100% *Aedes albopictus* suitability and the average number of extrinsic generations of the parasite within the vector: continental USA (**A**), Alaska (**B**), and Hawaii (**C**)

After habitat suitability models were generated for *Ae. albopictus* and *Ae. vexans* with different contribution percentages (0–100, 10–90, 25–75, 50–50, 75–25, 90–10, 100–0), they were weighted with the average number of generations of *D. immitis*, resulting in seven maps of potential risk of infection by *D. immitis* in the USA. In all risk maps, a positive and highly significant correlation was obtained between our predicted risk values and the number of cases reported in each state, confirming the predictive capacity of the models (Fig. 5, Table 2). Although all maps showed a positive and highly significant correlation, the optimal model was selected by jointly evaluating several criteria: *R*^2^, AIC, and residual variance. According to these statistics (together with the *P*-value), the map with 100% contribution from *Ae. albopictus* (*β* ± SE = 61.15 ± 16.39, *R*^2^ = 0.594, *P* < 0.001) proved to be the one that best fit the data and, therefore, the one that best represented the average annual risk of *D. immitis* infection in the USA (Fig. 6).

**Table 2:** Linear regression results for the different risk maps

Contribution (%)	Slope (*β*)	Standard error	*P*-value	*R*^2^	AIC	Residual deviance
100A	48.589	5.795	5.82 × 10 ^−11^ (***)	0.594	453.1	22,375
10V90A	48.848	5.844	6.36 × 10 ^−11^ (***)	0.593	453.3	22,456
25V75A	49.229	5.919	7.32 × 10 ^−11^ (***)	0.590	453.6	22,586
50V50A	49.845	8.755	9.50 × 10 ^−11^ (***)	0.586	454.1	22,829
75V25A	50.431	8.937	1.28 × 10 ^−10^ (***)	0.581	454.7	23,108
90V10A	50.765	9.050	1.55 × 10 ^−10^ (***)	0.577	455.1	23,293
100 V	50.981	9.127	1.78 × 10 ^−10^ (***)	0.575	455.4	23,424

Slope (*β*) represents the regression coefficient, standard error its associated error, *R*^2^ the coefficient of determination, AIC the Akaike information criterion, residual deviance the model’s deviance, A *Aedes albopictus*, V *Ae. vexans*

Significance codes: *** *P* < 0.001

The areas with a very high risk of infection by *D. immitis* are mainly located in the southeastern United States, with the highest incidence along the Gulf Coast (Texas, Louisiana, Mississippi, Alabama, and Florida) and extending toward the Atlantic in Georgia and South Carolina (Fig. 6). Hawaii also showed very high risk. Other high-risk areas are located along the eastern edge of the country, extending along the Atlantic Coast and the Mississippi Valley. Inland regions, such as the Midwest and areas of the Great Plains, have medium risk values, while in the northernmost latitudes (Rocky Mountains, Pacific Northwest, Great Lakes, and Alaska), the risk of infection is low to very low.

### Monthly infection risk maps for dirofilariasis

**Fig. 7:**
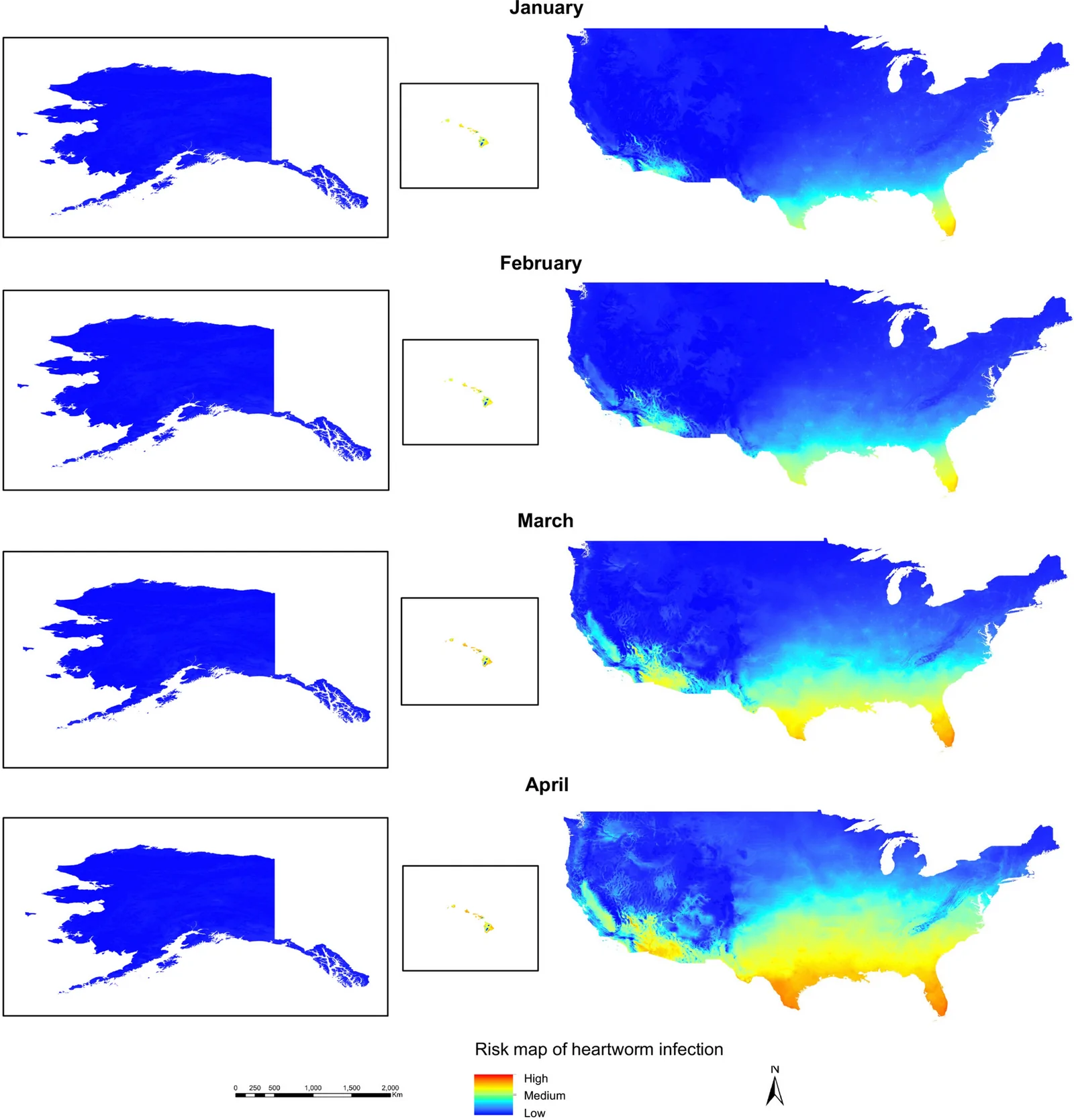
Monthly infection risk maps for *Dirofilaria immitis* in the USA from January to April, generated by combining the ecological niche model of 100% *Aedes albopictus* suitability with the estimated monthly number of extrinsic parasite generations

**Fig. 8:**
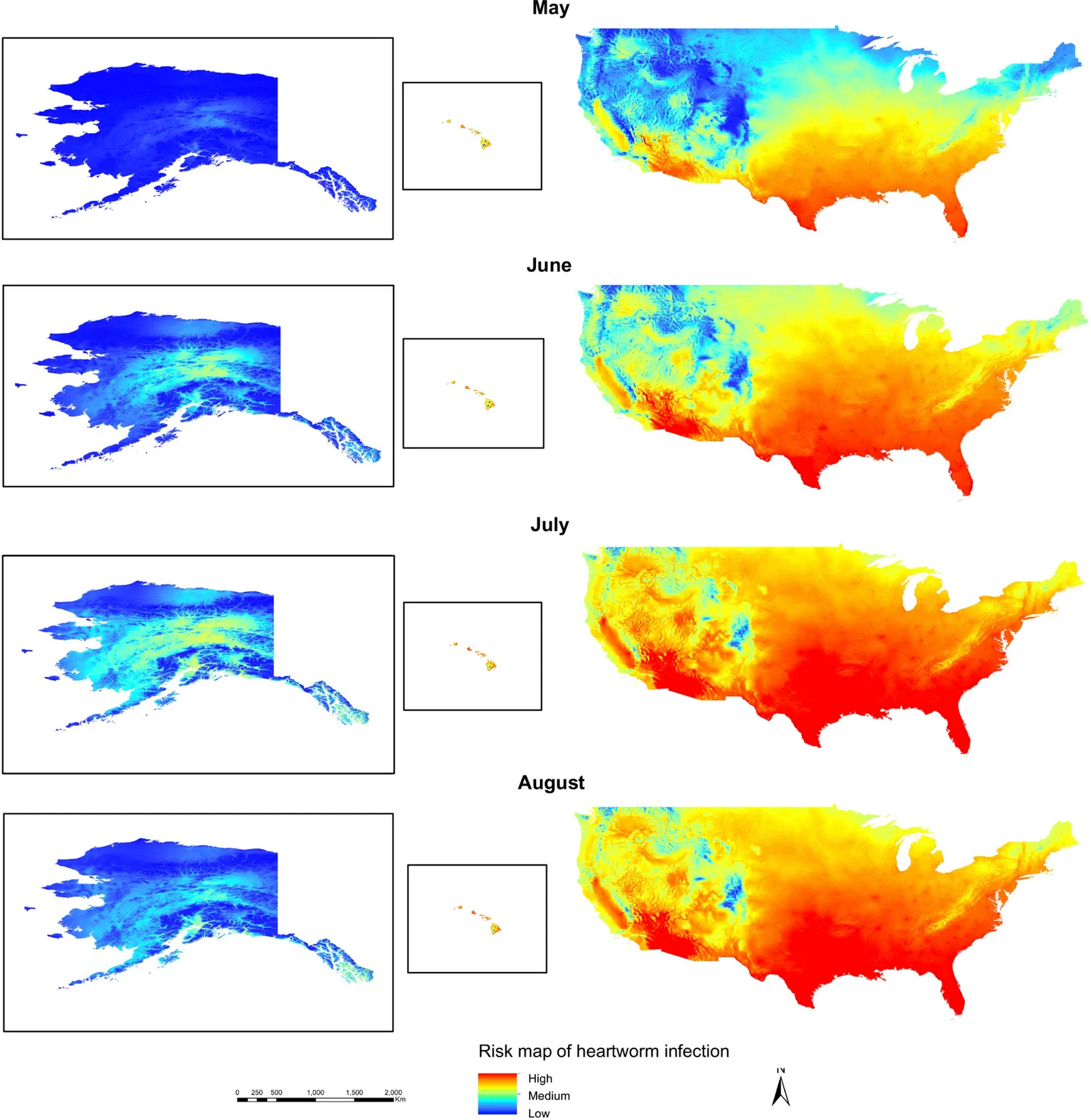
Monthly infection risk maps for *Dirofilaria immitis* in the USA from May to August, generated by combining the ecological niche model of 100% *Aedes albopictus* suitability with the estimated monthly number of extrinsic parasite generations

**Fig. 9:**
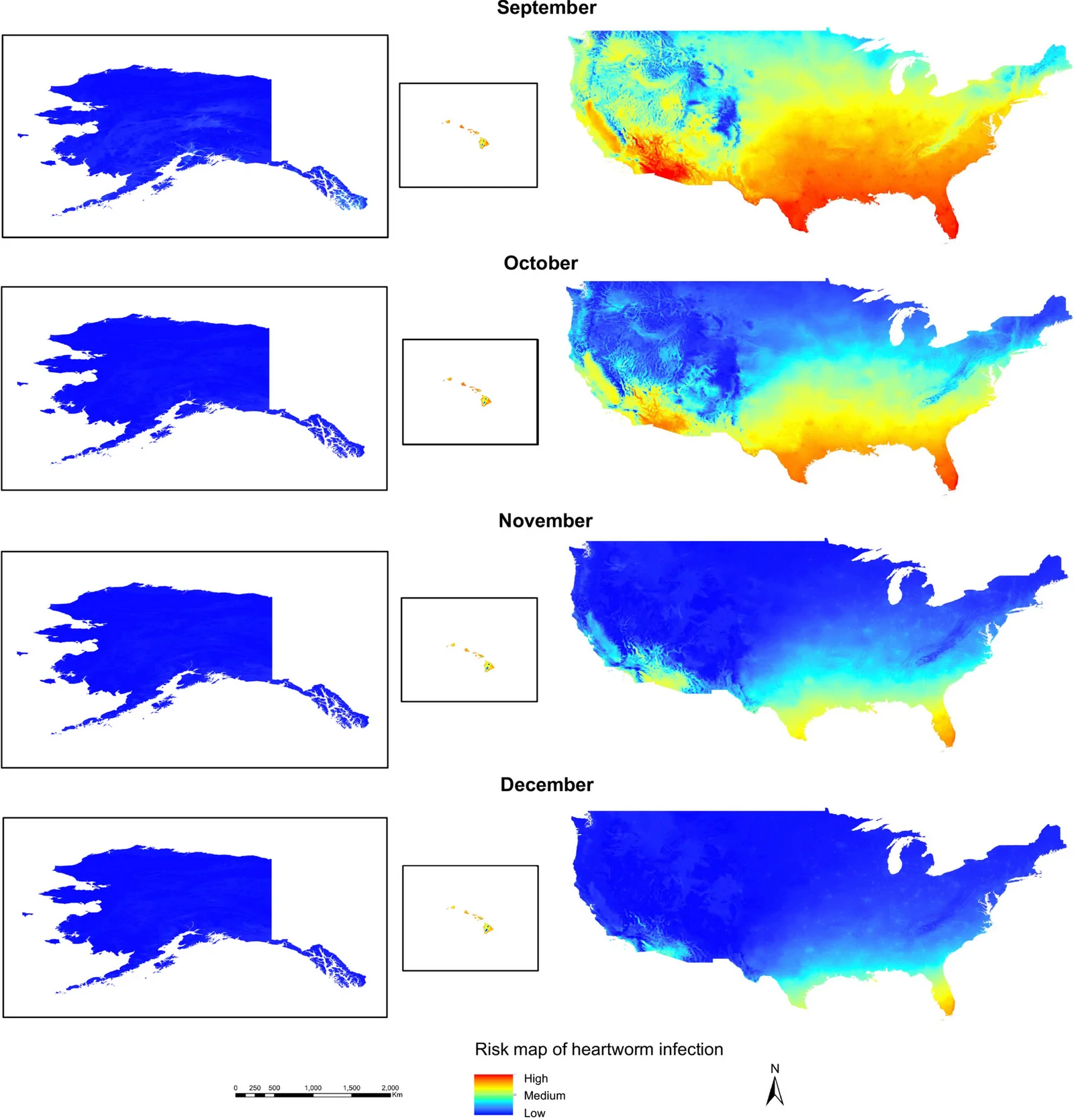
Monthly infection risk maps for *Dirofilaria immitis* in the USA from September to December, generated by combining the ecological niche model of 100% *Aedes albopictus* suitability with the estimated monthly number of extrinsic parasite generations

Monthly risk maps for the United States showed a marked seasonal pattern, with latitudinal variations throughout the year (Figs. 7, 8, 9). During the winter, the risk of infection remains very low in most of the country, limited to southern areas such as Florida and the Gulf Coast. In spring, the risk increases progressively from south to north, reaching average values in the southeastern and parts of the central USA. In summer, the risk reaches its maximum extent, with very high values in the southern states and the Mississippi Valley, and high levels in much of the central and eastern part of the country, while mountainous and higher-latitude regions remain at moderate/low levels. Finally, in fall, the risk gradually decreases from north to south, with transmission again restricted to the southernmost areas by the end of the season.

### Projection of habitat suitability for vectors in 2100

**Fig. 10:**
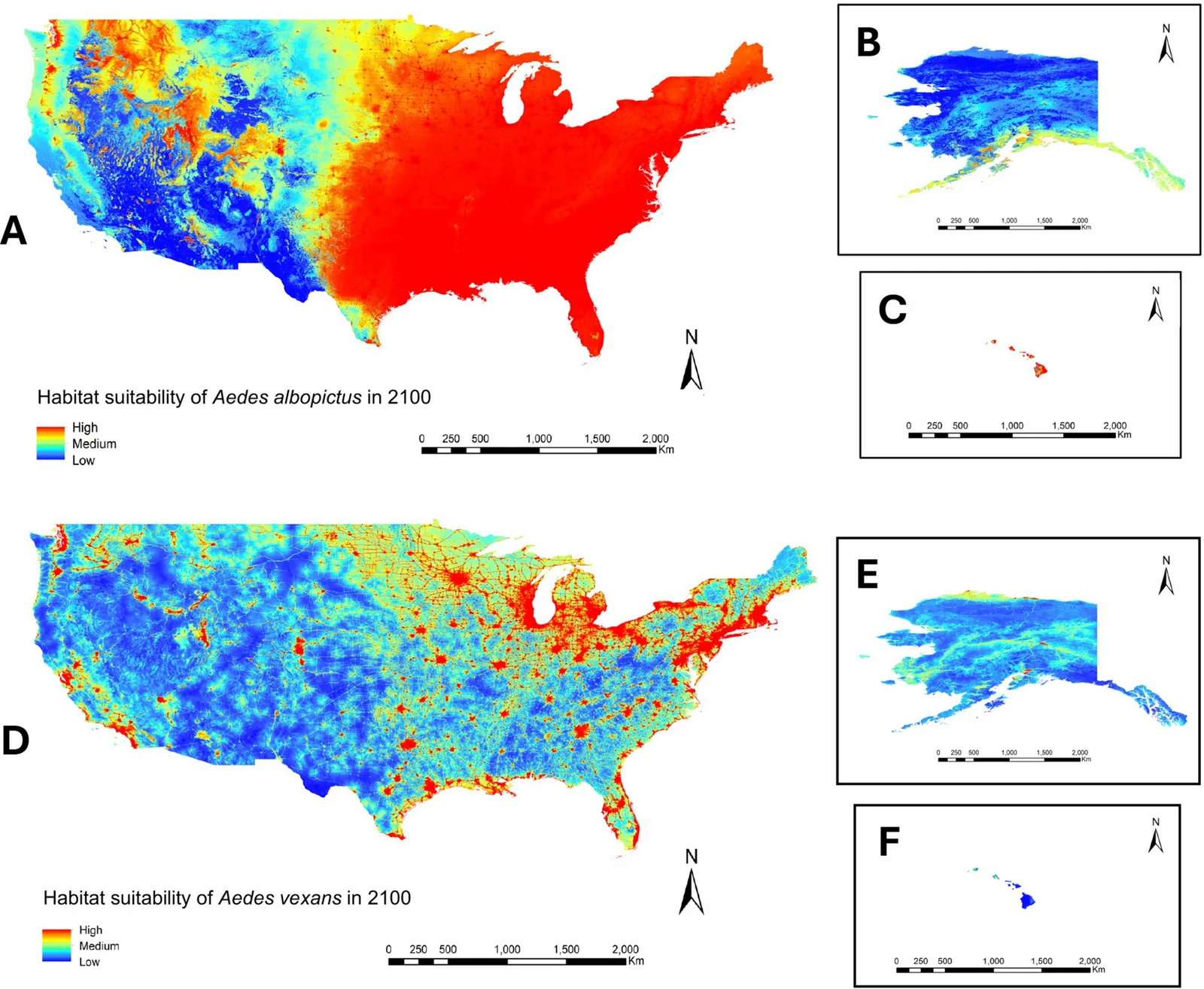
Projected habitat suitability for *Aedes albopictus* and *Aedes vexans* in the USA under the RCP 8.5 climate change scenario. **A**–**C** correspond to *Ae. albopictus* in the continental USA (**A**), Alaska (**B**), and Hawaii (**C**); **D**–**F** correspond to *Ae. vexans* in the continental USA (**D**), Alaska (**E**), and Hawaii (**F**)

The projection of the habitat suitability of *Ae. albopictus* for 2100 under a climate change scenario revealed a 123.78% gain in habitat suitability and a 2.36% loss. For *Ae. vexans*, the projection showed an increase of 21.75% and a loss of 19.11% in habitat suitability (Fig. 10). According to the range change analysis (Supplementary Fig. 4), the increase for *Ae. albopictus* is mainly concentrated in the northeastern regions of the country, as well as in areas of higher latitude and altitude that currently have cold climates and low suitability for this species. In contrast, with regard to the projection for *Ae. vexans*, gains in suitability in the north-central regions and Alaska are offset by losses in southern areas, suggesting a latitudinal shift in the vector's habitat suitability toward more northern areas.

## Discussion

Previous work in Europe has demonstrated that combining ecological suitability models with temperature-based estimates of *D. immitis* development is a robust approach to predicting heartworm transmission risk across diverse climatic regions [7, 56–65]. Recent studies have successfully applied integrated vector–parasite risk maps in Spain, Greece, Portugal, Serbia, and Italy [29–31, 62, 65], confirming the predictive value of this modeling framework. In contrast, heartworm risk in the United States has only been partially explored in three studies: one estimating transmission season length from temperature data alone [66], another constructing a county-level prevalence map based on empirical test results [67], and a third analyzing infection in coyotes using environmental predictors [68]. To our knowledge, this is the first nationwide study in the USA to integrate mosquito vector suitability with *D. immitis* extrinsic development, applying a methodology already validated in Europe.

Our modeling framework integrates vector habitat suitability with parasite thermal development to generate seasonally resolved risk maps for *D. immitis* across the USA. The selected MaxEnt configurations for *Ae. albopictus* (AUC = 0.786) and for *Ae. vexans* (AUC = 0.804) performed within the range typically reported for presence-only ENMs using heterogeneous occurrence data [41, 48]. Crucially, model validation against independent surveillance [10] yielded a strong positive relationship between predicted risk and state-level case counts (best model *Ae. albopictus* 100%: *R*^2^ = 0.594; *P* < 0.001), suggesting that our combined ENM–GDD approach retains epidemiological signal beyond purely environmental suitability.

The dominance of human footprint in both vectors—particularly the 87.1% contribution for *Ae. vexans*—is consistent with an extensive literature strand linking *Aedes* proliferation to urbanization, anthropogenic water storage, and built infrastructure [69, 70]. For *Ae. albopictus*, high weights for shrub density and annual mean temperature (BIO_1_) match its well-documented affinity for vegetated peri-urban habitats and warm conditions that shorten larval development and gonotrophic cycles [71]. The resulting suitability patterns—*Ae. albopictus* concentrated in the eastern and southeastern USA, and *Ae. vexans* broadly suitable across central–eastern regions with strong metropolitan associations—parallel prior national and regional maps of *Aedes* distribution [70, 72, 73].

By coupling suitability with extrinsic development thresholds for *D. immitis* (≥ 130 GDD above 14 °C), our risk maps recover the expected latitudinal and seasonal gradients: a clear Gulf Coast hotspot with an average of > 6 generations annually and a July peak exceeding a total of nine generations in the warmest areas, contrasted with near-null potential in Alaska. This agrees with classical and contemporary degree-day analyses showing temperature-limited transmission windows for heartworm and other filarioids [7, 8] and supports the mechanistic plausibility of using GDD as a bridge between vector suitability and parasite force of infection [74, 75]. The monthly progression from minimal risk in January to a July maximum mirrors veterinary observations of seasonal transmission pressure and prophylaxis recommendations across the southeast [10, 11].

The best-performing annual risk surface was the *Ae. albopictus*–driven 100% map. Two non-exclusive explanations align with published evidence. First, *Ae. albopictus* has expanded rapidly and established dense populations in the East, increasing contact with domestic dogs and peri-urban wildlife reservoirs [69, 70]. Second, *Ae. albopictus* often dominates container-breeding niches in cities and suburbs where veterinary care and testing are also more frequent, which can strengthen correlations between predicted risk and reported cases [73]. By contrast, *Ae. vexans*, while a competent, widespread floodwater mosquito, shows more ephemeral dynamics tied to hydrometeorology [76], which may dilute its direct contribution to annualized, state-level case metrics. Notably, all weighting schemes were highly significant, indicating that both vectors may contribute to spatial risk patterns.

The annual heartworm risk map highlights a strong spatial concentration of transmission in the southeastern USA, but the monthly risk maps reveal key temporal dynamics that are masked at annual scale. Risk begins to emerge in late winter in the Gulf states, expands northward during spring following temperature-driven increases in extrinsic parasite development, and reaches peak expansion in July–August, coinciding with optimal mosquito abundance and rapid L_3_ maturation. This seasonal pulse in transmission suitability follows a clear latitudinal gradient and contrasts with minimal winter risk, reinforcing the fact that heartworm is a strongly climate-dependent vector–parasite system. These findings agree with the temperature-forced transmission cycles proposed by Genchi et al. [7] in Europe and with seasonal veterinary data reported by the AHS [10]. Importantly, risk persists for up to 9–10 months in the Gulf Coast but is limited to 3–4 months in northern states, suggesting that expanding prophylaxis recommendations beyond the traditional “mosquito season” may be warranted in warmer regions under a One Health preventive framework.

Future projections under a high-emission scenario indicate substantial gains for *Ae. albopictus* (+123.78% suitable cells) and a more redistributive pattern for *Ae. vexans* (gains in north-central states and Alaska offset by southern losses). The pronounced northward and upslope expansion of *Ae. albopictus* produces a recurring signal in climate suitability studies for this species in temperate regions [70, 77]. For heartworm, this suggests that areas historically deemed marginal—parts of the Midwest, Great Lakes, and the Northeast Corridor—may experience longer transmission seasons and higher infection pressure by mid- to late-century, particularly in urban/suburban belts where the human footprint is high. In contrast, *Ae. vexans* appears more sensitive to shifting hydrological regimes; the projected south-to-north displacement is consistent with models showing climate-driven changes in floodplain dynamics and snowmelt that reconfigure its breeding habitats [76].

From a One Health perspective, three implications follow. First, risk is not static: veterinary protocols and vector control should incorporate climate-responsive timing and geography, prioritizing Gulf and southeastern states now while preparing for emerging risk corridors in the Midwest and Northeast. Second, the dominant effect of the human footprint underscores the fact that urban planning (stormwater management, container reduction, green infrastructure) and pet owner behavior (year-round prophylaxis, testing compliance, movement of dogs from high-incidence shelters) are as critical as climate in shaping future heartworm dynamics [10, 78]. Third, integrating wildlife hosts into surveillance—particularly peri-urban canids and mesocarnivores—may improve early warning where domestic dog testing lags [1, 8].

Our study also shares limitations commonly acknowledged in the field. Presence-only occurrence (GBIF) can retain sampling bias despite spatial thinning; MaxEnt infers suitability, not abundance; and state-level AHS data may reflect heterogeneous testing and reporting practices. Climate projections rely on a single global climate model and scenario; although HadGEM3-GC31-LL performs well for large-scale patterns, ensemble approaches would better capture uncertainty [51, 53]. Finally, translating suitability and GDD accumulation into actual force of infection would benefit from explicit vector bionomics (biting rates, parity), microclimate, and preventive coverage.

Despite these caveats, by fusing vector ENMs with parasite thermal biology at monthly resolution, we provide actionable risk stratification that can guide targeted prophylaxis, shelter dog screening policies, and community vector control—now and under future warming. Embedding these outputs within a One Health surveillance framework will help anticipate shifts in transmission suitability and mitigate the expanding burden of heartworm in the USA.

## Conclusions

This study provides the first nationwide, fine-scale prediction of *D. immitis* transmission risk in the USA integrating ENM of mosquito vectors with parasite development dynamics under current and future climate conditions. Our results reveal clear spatial and seasonal patterns of heartworm infection risk, with the highest suitability concentrated in the southeastern states but with notable expansion towards the Midwest and Northeast regions, particularly in summer and under future climate projections. These findings highlight the increasing relevance of vector-borne parasitic diseases in temperate regions and emphasize the need to incorporate climate-based surveillance tools into veterinary and public health strategies. The risk maps generated in this study offer a valuable decision-support tool for One Health planning, targeted prevention campaigns, and optimizing prophylactic measures in both endemic and emerging areas.

## Supplementary Information

Additional file 1: **Figure S1.** Prediction of the monthly number of generations of *Dirofilaria immitis* in the USA from January to April

Additional file 2: **Figure S2.** Prediction of the monthly number of generations of *Dirofilaria immitis* in the USA from May to August

Additional file 3: **Figure S3.** Prediction of the monthly number of generations of *Dirofilaria immitis* in the USA from September to December

Additional file 4: **Figure S4.** Range shift analysis showing areas of habitat suitability loss, gain, and stability for *Aedes albopictus* and *Aedes vexans* across different US territories. Panels A–C correspond to *A. albopictus* in the continental USA (A), Alaska (B), and Hawaii (C); panels D–F correspond to *A. vexans* in the continental USA (D), Alaska (E), and Hawaii (F).

## Data Availability

The datasets supporting the conclusions of this article are included within the article.

## Abbreviations

*Ae. Albopictus*: *Aedes* *albopictus*
*Ae. vexans*: *Aedes vexans*
AUC: Area under the curve
BIO_1_: Annual mean temperature
BIO_2_: Mean diurnal range
BIO_8_: Mean temperature of wettest quarter
BIO_9_: Mean temperature of driest quarter
BIO_12_: Annual precipitation
BIO_15_: Precipitation seasonality
BIO_18_: Precipitation of warmest quarter
BIO_19_: Precipitation of coldest quarter
*Cx. pipiens*: *Culex pipiens*
*D. immitis*: *Dirofilaria immitis*
ENM: Ecological niche model
ENMa: Habitat suitability model for *Ae. albopictus*
ENMv: Habitat suitability model for *Ae. vexans*
GDDs: Growing degree days
